# GHR knockdown enhances the sensitivity of HCC cells to sorafenib

**DOI:** 10.18632/aging.103625

**Published:** 2020-09-24

**Authors:** Shuang Gao, Qianwen Ni, Xiuli Wu, Tieliu Cao

**Affiliations:** 1 Department of Gastroenterology, The Third Affiliated Hospital of Naval Military Medical University, Shanghai 201800, China; 2 Department of Gastroenterology, Zhongshan Qingpu Hospital Fudan University, Shanghai 201799, China; 3 Department of Gastroenterology, Luoyang First People's Hospital, Luoyang 471000, China; 4 Department of Traditional Chinese Medicine, Minhang Branch, Shanghai Cancer Center, Fudan University, Shanghai 200240, China

**Keywords:** hepatocellular carcinoma, sorafenib, GHR, resistance, PI3K/AKT/ERK1/2 signaling pathway

## Abstract

Sorafenib is approved for treatment of advanced hepatocellular carcinoma (HCC) by the Drug Administration. However, the efficacy of sorafenib has become very limited because most tumors have developed resistance to this drug. In this study, we found that sorafenib stimulated GHR expression in HCC cell lines. Thus, GHR might be linked to sorafenib resistance. To verify this hypothesis, we researched the roles of GHR knockdown and sorafenib combination in cell viability, apoptosis, cycle, and migration. The results showed that GHR blockage enhanced sorafenib blocking of cell cycle progression, leading to inhibition of this drug on HCC cell viability, and the improved promoting ability of sorafenib on cell apoptosis. In addition, it was found that GHR knockdown enhanced sorafenib inhibition of cell migration. The synergistic antitumor effects of sorafenib and GHR knockdown combination may be attributed to inhibition of PI3K/AKT/ERK1/2 signaling pathway. In conclusion, the findings suggest that GHR knockdown enhances the sensitivity of HCC cells to sorafenib. and the inactivation of PI3K/AKT/ERK1/2 signaling pathway may be the underlying mechanisms. This highlights the absence of GHR as a promising way to enhance sorafenib efficacy in HCC.

## INTRODUCTION

Hepatocellular carcinoma (HCC), a leading cause of cancer-related death worldwide, tortures more than 840,000 people every year [1]. In recent years, the incidence of HCC is rising faster than that of any other cancers in both males and females [2]. China has the highest incidence of HCC, which accounts for more than a half of the world’s burden [3]. Patients with early HCC are usually treated with surgery and liver transplantation [4]. However, in for most patients, HCC is diagnosed at an advanced stage. In other words, these patients have missed the best treatment period. HCC is usually associated with liver function impairment, limiting the efficacy of chemotherapy [5]. Thus, the 5-years survival rate is still very low.

Sorafenib, an oral multi-kinase inhibitor, inhibits tumor angiogenesis and reduces tumor cell apoptosis [6]. In 2007, it is approved for the treatment of advanced HCC by the Drug Administration [7]. The mechanism underlying the therapeutic effects of sorafenib on HCC has been well studied. Sorafenib firstly targets multiple kinases which are involved in the Ras/Raf/MEK/ERK signaling pathway, such as Raf-1 and B-Raf, to directly suppress tumor cell proliferation [3]. In most advanced HCCs, the Ras/Raf/MEK/ERK signaling pathway is activated by the stimulation of growth factors, including epidermal growth factor (EGF), hepatocyte growth factor (HGF), and insulin-like growth factor (IGF) [8]. In addition, sorafenib indirectly suppresses tumor cell proliferation through targeting c-Kit, FLT-3, VEGFR-2/3, PDGFR-β, and other tyrosine kinases, which are involved in tumor angiogenesis [3]. Although the overall survival is improved in HCC patients treated with sorafenib, the median survival time of those with advanced disease is only about 3-5 months [8]. Previous studies have provided many evidences that the efficacy of sorafenib for patients with advanced HCC is very limited [9–11]. Most of those patients develop resistance to this drug, which has become a limiting factor in its clinical application [12, 13]. Currently, various factors are reported to be connected with sorafenib resistance, such as EMT, drug metabolism, angiogenesis, hypoxia, autophagy, inflammation viral activation and the activation of signal pathways [3].

Growth hormone (GH), the main mediator of the postnatal growth of somatic cells, plays a critical role in cell growth and differentiation via interacting with its receptor (GHR) [14]. GHR belongs to a large family of cytokine or hematopoietic receptors. It activates signal transductors (JAK-2/STAT), the cascade of the mitogen-activated protein kinase (MAPK) and the phosphoinositide 3-kinase (PI3K) which are important for cell growth and survival [15]. Recently, GHR is reported to be associated with cancer development and progression, including breast cancer and hepatocellular carcinoma [16–18]. In addition, GHR is connected with breast cancer chemoresistance and metastasis that GHR knockdown decreases the chemoresistant and metastatic behavior of estrogen receptor negative breast cancer [19, 20].

In this study, it was found that sorafenib could stimulate GHR expression in HCC cell lines, while other drugs including regorafenib, lenvatinib, and cabozantinib had no effects on GHR expression. Therefore, we further detected the impacts of GHR knockdown and sorafenib combination on cell viability, apoptosis, cycle, and migration. The results showed that GHR silence sensitized HCC cells to sorafenib. The inactivation of PI3K/AKT signaling pathway and ERK1/2 might be the underlying mechanisms, highlighting the absence of GHR as a promising way to enhance sorafenib efficacy in HCC.

## RESULTS

### Sorafenib induced the increase of GHR in HCC cell lines

**Figure 1:**
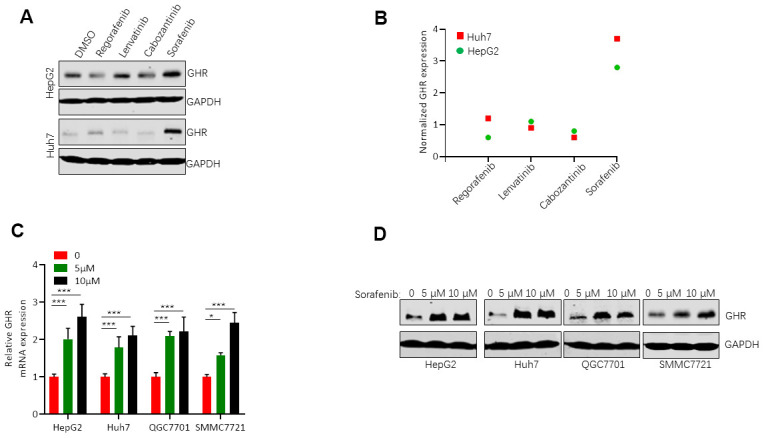
**Sorafenib induced GHR expression in HCC cell lines.** (**A**) Western blotting assay measured the protein levels of GHR in HepG2 and Huh7 cell lines treated with sorafenib, regorafenib, lenvatinib, or cabozantinib; (**B**) The quantification of GHR protein expression was normalized by Image J; (**C**) The mRNA levels of GHR were detected in different HCC cell lines with the treatment of 0, 5 μM or 10 μM sorafenib by Real-time PCR assay. (**D**) Western blotting assay detected the protein levels of GHR in different HCC cell lines treated with 0, 5 μM or 10 μM sorafenib.

GHR is highly expressed in tumor samples compared with adjacent normal tissues [15], and it regulates tumor cell proliferation, apoptosis, tumor differentiation and tumor grade [21, 22]. In this study, we first detected the relationship between GHR expression and drugs used in the treatment of HCC. The results of western blotting assay showed that sorafenib stimulated GHR expression in HepG2 and Huh7 cell lines, while other drugs including regorafenib, lenvatinib, and cabozantinib had no effects on GHR expression in HCC cell lines (Figure 1A, 1B). To further validate the impact of sorafenib on GHR expression in HCC cell lines, the mRNA level of GHR was increased in different HCC cell lines including HepG2, Huh7, QGC7701 and SMMC7721 cells treated with 5 μM or 10 μM sorafenib. (Figure 1C). In addition, the protein expression of GHR was also highly increased in these four types of HCC cell lines treated with different concentration of sorafenib (Figure 1D). These findings suggested that GHR might play a role in sorafenib treatment for HCC.

### GHR blockage increased sorafenib inhibition of HCC cell viability

**Figure 2:**
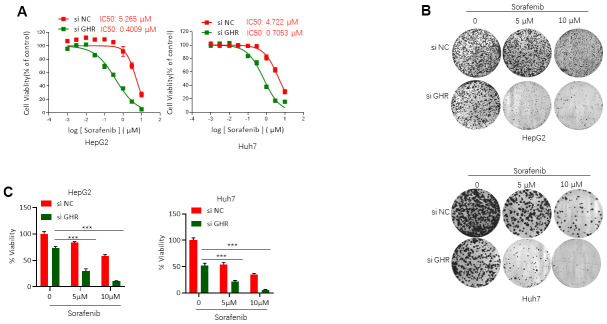
**GHR blockage increased sorafenib inhibition of HCC cell viability.** (**A**) MTT assay tested the IC50 values of sorafenib on HepG2 and Huh7 cells with the presence or absence of GHR. In HepG2 cells with siGHR, IC50 values is 0.4009 μM, whereas on control cells the value is 5.265 μM. In Huh7 cells with siGHR, IC50 values is 0.7053 μM, and on control cells the value is 4.722 μM; (**B**) The results of colony formation assay showed that GHR silence significantly inhibited cell proliferation in HCC cells treated with different concentration of sorafenib (0, 5 μM or 10 μM); (**C**) The percentage of cell viability was normalized. All data shown represented the mean ± SEM. ***P < 0.001, compared with control groups.

To gain insight into the functional role of GHR in sorafenib resistance for HCC treatment, si GHR was transfected into two HCC cell lines, HepG2 and Huh7, for silencing GHR. Next, we analyzed the effects of sorafenib on the cell viability of these cells with MTT assay. IC50 of sorafenib on HepG2 and Huh7 cells transfected with si GHR or si RNA were detected. Cell viability was decreased in a dose-dependent manner when cells were treated with sorafenib (Figure 2A). Sorafenib showed 50% viability inhibition on HepG2 cells with GHR blockage at 0.4009 μM, presenting a 13-fold lower IC50 value than that on HepG2 cells (IC50 = 5.265 μM). In addition, the IC50 value of sorafenib on Huh7 cells transfected with siGHR is 0.7053 μM, whereas for control cells the value is 4.722 μM. These findings suggest that GHR inhibition induces sensitization of HCC cells to sorafenib.

To strengthen these observations, colony formation assay was further performed. Notably, GHR knockdown aggravated the inhibition effects of sorafenib on the cell proliferation of HCC cells (Figure 2B, 2C).

### GHR knockdown enhanced sorafenib suppressing of the activation of PI3K/AKT/ERK1/2 signaling pathways

**Figure 3:**
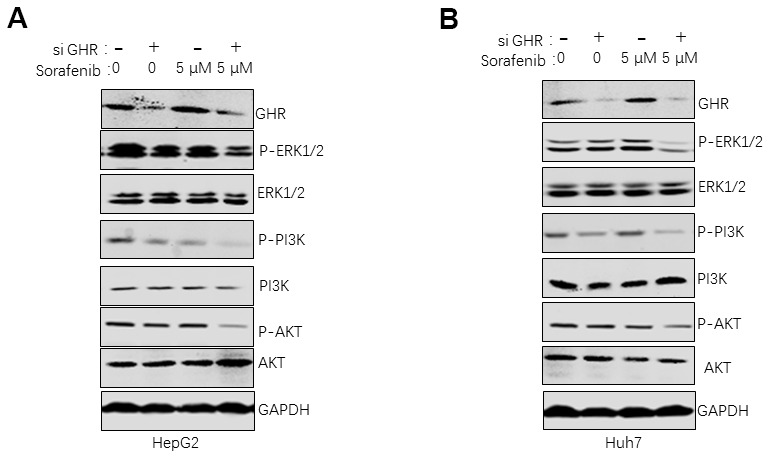
**GHR knockdown enhanced sorafenib suppressing of the activation of PI3K/AKT/ERK1/2 signaling pathways.** The results of western blotting assay revealed that the protein levels of ERK1/2, p-ERK1/2, PI3K, p-PI3K, AKT and p-AKT were significantly inhibited in HepG2 (**A**) and Huh7 cells (**B**) with siGHR and sorafenib combination compared with cells with sorafenib single.

To investigate the underlying mechanism of GHR silence sensitizing HCC cells to sorafenib, this study detected molecules of ERK1/2, and PI3K/AKT signaling pathways in HepG2 and Huh7 cells after si GHR and drug treatment by using western blotting assay. HCC cells were divided into four groups according to the treatment with or without sorafenib or si GHR. It is important to highlight that the protein levels of p-ERK1/2, p-PI3K and p-AKT were significantly lower in HCC cells treated with both si GHR and sorafenib than the other three groups (Figure 3A, 3B), suggesting that GHR knockdown induced the inhibition effects of sorafenib on the activation of PI3K/AKT/ERK1/2 signaling pathways.

### GHR knockdown enhanced sorafenib promoting of cell apoptosis and blocking of cell cycle progression

**Figure 4:**
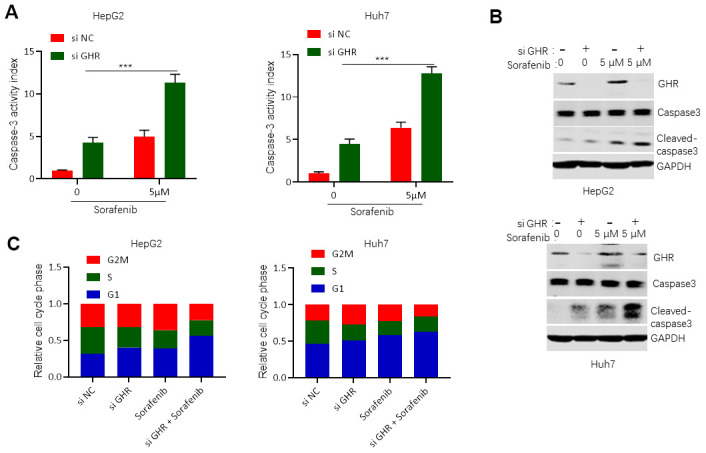
**GHR knockdown enhanced sorafenib promoting of cell apoptosis and blocking of cell cycle progression**. (**A**) Caspase-3 activity assay detected caspase-3 activity in HCC cell lines with siGHR or/and sorafenib; (**B**) The results of western blotting assay showed that the protein levels of cleaved caspase-3 were significantly increased in HCC cell lines with siGHR and sorafenib combination compared with cells with sorafenib single; (**C**) GHR inhibition combined with sorafenib prevented HCC cell progression from G stage to S and G2M stages. All data shown represented the mean ± SEM. ***P < 0.001, compared with control groups.

Caspase-3, a cysteine protease, plays an important role in execution of apoptosis. The proteolytic cleaves pro-caspase-3, an inactive proenzyme, into its active form, called cleaved caspase-3, which is responsible for specific cleavage of large numbers of key cellular proteins involved in apoptosis [23]. In this study, we detected the role of GHR knockdown and sorafenib in caspase-3 activity. The results showed that silencing GHR or sorafenib treatment single promoted caspase-3 activity and the protein level of cleaved caspase-3 compared with control group (Figure 4A, 4B). However, caspase-3 activity and cleaved caspase-3 protein level were significantly higher in HCC cells treated with both si GHR and sorafenib than that of cells treated with single. These findings suggest that GHR knockdown aggravated the promotion function of sorafenib on cell apoptosis of HCC cells.

To investigate whether si GHR combined with sorafenib-induced inhibition of cell growth was connected with cell cycle dysregulation, we further tested cell cycle of HCC cells when treated with si GHR or/and sorafenib. Silencing GHR or/ and sorafenib treatment stimulated HepG2 and Huh7 accumulation in G1 stage, while cell growth was then significantly reduced in S stage (Figure 4C). Interestingly, only si GHR combined with sorafenib treatment continued cell growth decrease from S stage to G2M stage, suggesting that GHR inhibition combined with sorafenib prevented HCC cell progression from G stage to S and G2M stages.

### GHR knockdown enhanced sorafenib inhibiting of cell migration

**Figure 5:**
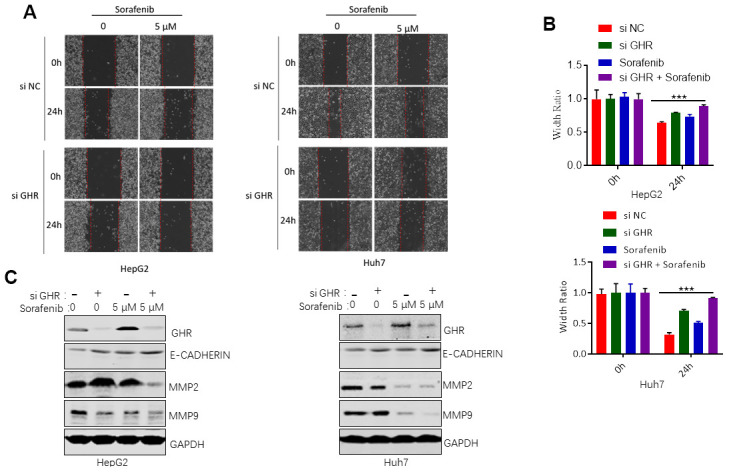
**GHR knockdown enhanced sorafenib inhibiting of cell migration.** (**A**) Wound healing assay measured cell migration of HCC cells siGHR or/and sorafenib; (**B**) the width ratio was calculated by the wound width/the distance measured at 0 h. All data shown represented the mean ± SEM. ***p<0.001; (**C**) The results of western blotting assay showed that GHR knockdown and sorafenib combination suppressed the protein levels of MMP2 and MMP9 and promoted the expression of E-cadherin in HepG2 and Huh7 cells.

Next, we further investigated the roles of si GHR or/and sorafenib in HCC cell migration. Wounding healing assay results revealed no significant difference between the cell migration area of HepG2 and Huh7 cells treated with si GHR or sorafenib individually compared with that of the control cells in 24 h (Figure 5A, 5B). Combination treatment with si GHR plus sorafenib significantly increased the wound size of HepG2 and Huh7 cells. In addition, we also evaluated the protein levels of MMP2 and MMP9 in HCC cells treated with si GHR or/and sorafenib. Matrix metalloproteinases (MMPs) have been reported to promote cell migration and invasion via extracellular matrix (ECM) degradation, resulting in metastasis [24]. MMP2 and MMP9, the major proteolytic enzymes contributed to ECM degradation, are involved in the process of releasing vascular endothelial growth factor (VEGF), promoting tumor cell migration and invasion [24]. Western blotting assay results showed that sorafenib single inhibited MMP9 expression in HepG2 cells and suppressed both MMP2 and MMP9 expression in Huh7 cells (Figure 5C). Except that, loss of E-cadherin gene expression causes dysfunction of cell junction system, allowing cancer cell invasion and metastasis. To further validate the underlying mechanism by which GHR knockdown and sorafenib combination regulates E-cadherin, we found that GHR knockdown and sorafenib combination had an increased expression of E-cadherin at protein levels (Figure 5C), These results suggested that the combination treatment notably proved the roles of GHR blockage in sorafenib inhibiting of cell migration.

## DISCUSSION

GHR, a member of the class I cytokine receptor family, plays a critical role in cancer development, and is associated with cancer chemoresistance as well as metastasis [20, 25]. In this study, we found that GHR expression was increased in HCC cell lines induced by sorafenib compared with cells without sorafenib treatment. Sorafenib, a small molecule, is well known for inhibiting tumor angiogenesis and promoting tumor cell apoptosis [6]. It is approved for treatment of advanced HCC by the Drug Administration in 2007 [7]. However, most advanced HCCs obtain resistance to sorafenib, eventually leading to tumor growth or distant metastasis overtime [8]. In this study, we investigated the roles of GHR blockage in sorafenib resistance in HCC development.

In detail, we found that GHR knockdown enhanced sorafenib inhibiting of cell viability. The IC50 value of sorafenib on HepG2 and Huh7 cells transfected with si GHR was significantly lower than that on control cells. In addition, GHR blockage increased the promotion effects of sorafenib on cell apoptosis. The functions of GHR inhibition and sorafenib combination on cell growth and apoptosis might result from the prevention of cell cycle progression. This study further found that GHR knockdown enhanced sorafenib inhibiting of cell migration. These findings suggest that GHR inhibition sensitizes HCC cells to sorafenib, which may be used as an effective strategy to suppress the resistance of HCC on sorafenib in clinical.

HCC development and progression mainly involve the Ras/Raf/MAPK and the PI3K/AKT/mTOR pathways [26]. Sorafenib, a multi-kinase inhibitor, targets VEGFR, PDGFR, PI3K, MAPK, c-kit, and Raf [27]. Previous studies have demonstrated that sorafenib resistance in HCC is regulated by several signaling pathway, including EGFR signaling, PI3K/AKT pathway, autophagy and Epithelial-mesenchymal transition (EMT) [4]. The PI3K/AKT signaling pathway plays a critical role in the signal transduction activities related to cell proliferation, apoptosis, and metastasis in human malignancies [28], and has an important impact on modulating sorafenib resistance in HCC [29]. AKT inhibitor MK-2206 and AKT knockdown have been reported to reverse the resistance to sorafenib [30]. In addition, AKT silence enhances the sensitivity of HCC cells to sorafenib-induced apoptosis [31]. The mechanism of PI3K/Akt pathways sorafenib resistance has been attracting attention. Sorafenib activates AKT and upregulates the phosphorylation of its downstream targets, including S6K, which is aberrantly activated in 40%–50% of HCC patients [32, 33]. Activated S6K causes crosstalk on the Ras/Raf/MAPK signaling pathway, and further diminishes the inhibitory effect of sorafenib on HCC, leading to sorafenib resistance to HCC [32, 34]. Furthermore, PI3K/AKT cascade is demonstrated to directly or indirectly regulate the properties of migration and invasion in sorafenib-resistant HCC cells through MMP2 and MMP9 [35]. In the present study, the protein levels of p-PI3K, p-AKT, MMP2 and MMP9 were significantly lower in HCC cells treated with both si GHR and sorafenib than that in control cells, suggesting that GHR knockdown induced the inhibition effects of sorafenib on the activation of PI3K/AKT signaling pathways. GHR activates signal transductors (JAK-2/STAT), the cascade of the mitogen-activated protein kinase (MAPK), and of the phosphoinositide 3-kinase (PI3K), which are important for cell growth and survival [15]. This study also found that GHR silence significantly inhibited the protein level of p-PI3K in HCC cells. Thus, GHR knockdown may enhance the sensitivity of HCC cells to sorafenib via dysregulating the activation of PI3K/AKT pathway.

In addition, our results showed that the protein level of p-ERK1/2 was also inhibited in HCC cells with si GHR and sorafenib combination in relative to control cells, suggesting ERK1/2 inactivation might be another mechanism of GHR silence sensitizing HCC cells to sorafenib. Previous studies reports that sorafenib response is impaired in HCC with dysregulated p-ERK activation [36], and overexpression of p-ERK1/2 leads to sorafenib resistance [37].

## CONCLUSIONS

Taken together, the present study showed that GHR knockdown enhanced the sensitivity of HCC cells to sorafenib, and the inactivation of PI3K/AKT/ERK1/2 signaling pathways might be the underlying mechanisms, highlighting the absence of GHR as a promising way to enhance sorafenib efficacy in HCC.

## MATERIALS AND METHODS

### Cell lines and cultures

Four HCC cell lines, including HepG2, Huh7, QGC7701, and SMMC7721 cells, were cultured in Dulbecco modified Eagle medium (DMEM; Gibco BRL, Grand Island, NY, USA) supplemented with 10% fetal bovine serum at 37°C in an atmosphere containing 5% CO2.

### Cell transfection

GHR knockdown cells were established by using siRNA which is directed against GHR. HepG2 and Huh7 cells were transfected with si GHR using Lipofectamine 2000 transfection reagent (Life Technologies, Grand Island, NY) in accordance with the manufacturer's instructions. First, cells were cultured in six well plates overnight. Then, 5 μg plasmids were added into each well mixed with lipofectamine solution.

### Western blotting assay

HepG2, Huh7, QGC7701, and SMMC7721 cells were treated with sorafenib, regorafenib, lenvatinib, or cabozantinib in the presence or absence of GHR for 24 h. Total protein was extracted from cells, and resolved on SDS-PAGE. Then, proteins were transferred to PVDF membranes. The membranes were further blocked with 5% skim milk for 2 h at room temperature, and then incubated overnight at 4°C with primary antibodies. Protein expression was examined using antibodies against GHR, GAPDH, p-ERK1/2, p-PI3K, p-AKT, cleaved-caspase 3, MMP2, and MMP9.

### Cell viability assay

MTT assay was performed to detect cell viability. Briefly, cells were cultured in a 96-well plate for 24 h, and then treated with different concentrations of sorafenib at 37°C for 24 h. Cells were incubated with 0.5 mg/mL MTT for 3 h. The result was measured spectrophotometrically with a microplate reader (multiplate reader multiskan FC, thermo scientific) at 570 nm.

### Colony formation assay

HCC cells with the presence or absence of GHR were cultured in 60-mm dishes containing a top layer of 0.7% agar and a bottom layer of 1% agar, and treated with 5 μM sorafenib for 4 weeks at 37°C. Last, cells were stained with 0.2% crystal violet.

### Caspase-3 activity assay

The caspase-3 activity was measured by using a caspase-3 activity assay kit (Beyotime, Shanghai, China) according to the manufacturer’s instructions. In brief, cells were cultured in 96-well plates and treated with sorafenib for 24 h. And then, cells were lysed with a lysis buffer (100 μl/well) for 15 min on ice. After washing with cold HBSS, cells were incubated with the mixture composed of a 10 μl cell lysate, 80 μl reaction buffer and 10 μl of 2 mM caspase 3 substrate at 37°C for 4 h. Last, the caspase 3 activity was measured using a SpectraMax M3 microplate reader (Molecular Devices) at an absorbance of 405 nm.

### Cell cycle analysis

Cell cycle was analyzed by using DNA flow cytometry. HCC cells with the presence or absence of GHR were treated with sorafenib for 24 h. And then, cells were fixed in 70% ethanol at 4°C, and treated with RNase, followed by staining with PI n the dark for 30 min. FACScan flow cytometer (Becton Dickinson, San Jose, CA) was performed to analyze cell cycle results.

### Wound healing assay

HCC cells with the presence or absence of GHR were transformed to 6-well plates, and treated with sorafenib. A 100 μl pipette tip was used to scrap the cells, when 80% of the well was covered with cells. After wounds generation, cells were incubated at 37°C for 24 h. The migration distance of cells was determined. The width ratio was calculated by the wound width/the distance measured at 0 h.

### Statistical analysis

All experiments were carried out in triplicate. The data are presented as the mean ± SEM. Statistical tests were performed using software SPSS 19.0. Student’s t-tests were performed to compare difference of the means between two groups. P values of <0.05 were considered as statistically significant.
